# Electrical coupling in the retina ganglion cell layer increases the dynamic range

**DOI:** 10.1186/1471-2202-15-S1-P59

**Published:** 2014-07-21

**Authors:** Cesar A Celis, Rodrigo Publio, Antonio C Roque

**Affiliations:** 1 Department of Physics, FFCLRP, University of Sao Paulo, Ribeirao Preto, Sao Paulo, Brazil

## 

**Figure 1:**
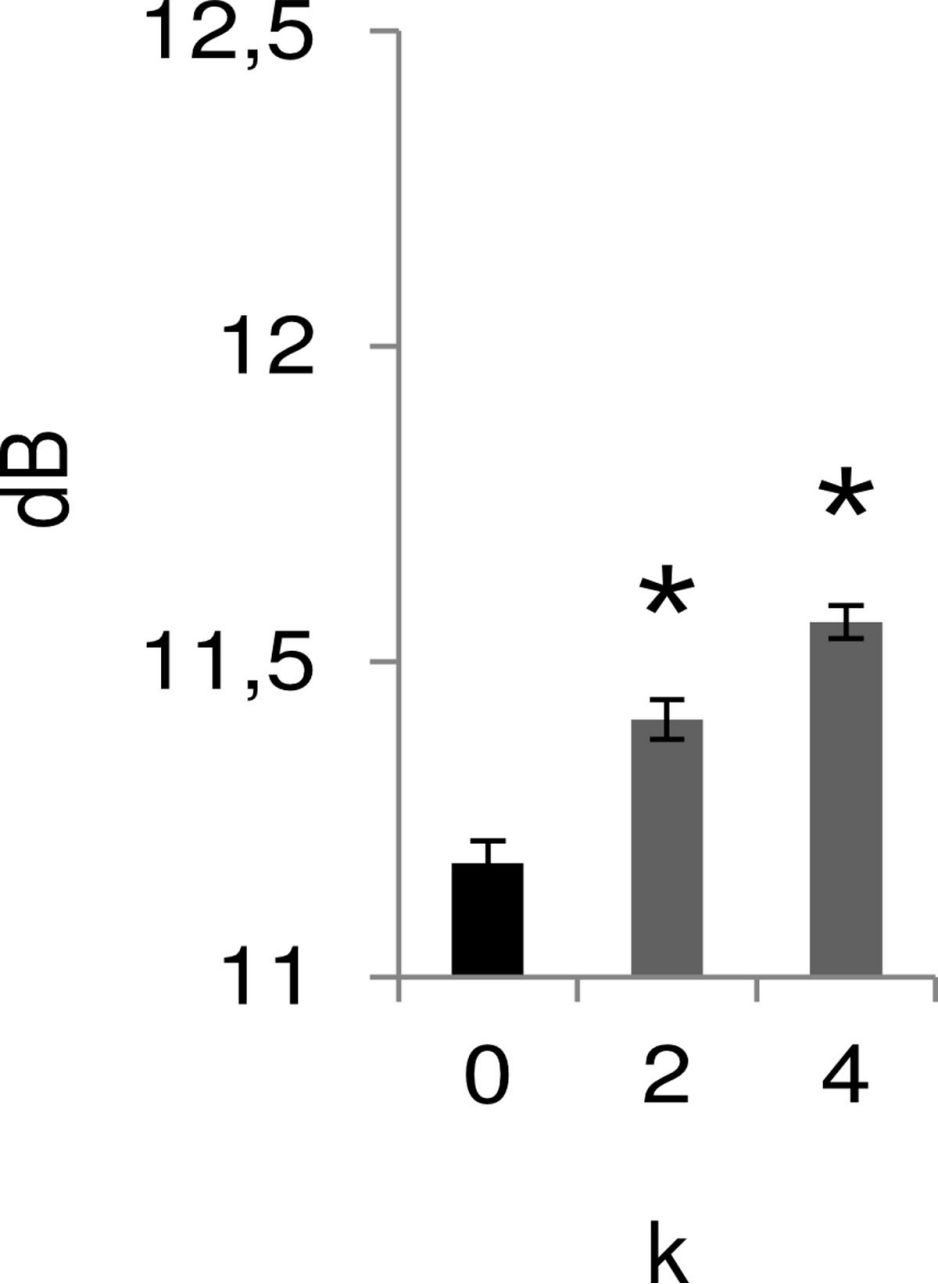
Effect of electrical coupling on the dynamic range of the GC network for different values of the connectivity index *k*. Each column corresponds to the mean value of 300 neurons. * Significantly different from the control group with *k* = 0 (Dunn, P < 0.05).

The vertebrates deal with visual stimuli with a broad range of intensity values. To enable these organisms to respond to this wide range of intensities there must be some mechanism capable of compressing the sensory signals at the retina [1]. It has been hypothesized that the wide dynamic range of the retina is due to electrical coupling of retinal ganglion cells (GCs) at the retina´s output layer [1-3]. The dynamic range of a network of neurons can be defined as the range of input stimuli values for which the network responds before saturation. In this work we constructed a biologically plausible model of the ganglion cell layer of the salamander retina and submitted it to simulated input stimuli. The GCs are connected via gap junctions and different values of the average connectivity were used to evaluate the effect of electrical coupling on the dynamic range of the network. We used the conductance-based model of the tiger salamander GC of Fohlmeister and Miller [4]. This is a single-compartment model containing the following voltage-dependent membrane conductances: sodium, calcium, A-type potassium, calcium activated potassium, and delayed rectifier potassium. A 20x20 square lattice was built with one GC in each node. Each GC in the network is randomly connected by electrical synapses with its nearest neighbors, with the average number of coupled neighbors being *k* = 0, 2 or 4. The network was stimulated by injecting in each cell an excitatory postsynaptic current with synaptic conductance modeled by an alpha function with decay time of 400 ms. The reversal potential of the synaptic current was set as 1.5 mV. The simulations were run using the NEURON simulator. The response of the network was taken as the total number of spikes during 1 sec of stimulation. Based on the F-I curves for the network we calculated the dynamic range (in decibels) for each average connectivity index *k*. The results (Figure 1) show that electrical coupling enhances the dynamic range of the GC network. This suggests that GC coupling via electrical synapses may be involved in the mechanism that endows the vertebrate retina with its large dynamic range.
